# Patient experiences of the urgent cancer referral pathway—Can the NHS do better? Semi‐structured interviews with patients with upper gastrointestinal cancer

**DOI:** 10.1111/hex.13136

**Published:** 2020-09-28

**Authors:** Anna Haste, Mark Lambert, Linda Sharp, Richard Thomson, Sarah Sowden

**Affiliations:** ^1^ Department of Psychology School of Social Sciences, Humanities and Law Teesside University Middlesbrough UK; ^2^ Public Health England North East Centre Newcastle UK; ^3^ Newcastle University Centre for Cancer Population Health Sciences Institute Newcastle University Newcastle upon Tyne UK; ^4^ Population Health Sciences Institute Newcastle University Newcastle upon Tyne UK

**Keywords:** cancer, patient experience, qualitative, service evaluation, urgent pathway

## Abstract

**Background:**

Timeliness is viewed as a key feature of health‐care quality. Internationally, this is challenging. In England, cancer waiting time targets are currently not being met. For example, between 2015 and 2018 only 71% of patients with upper gastrointestinal (UGI) cancer started treatment within the recommended 62 days of referral.

**Objective:**

We explored patients’ experiences to identify areas for service improvement.

**Design:**

Semi‐structured interviews were conducted.

**Setting and participants:**

Twenty patients who were referred through the urgent (two‐week) GP referral route and were within six months of receiving first treatment were recruited.

**Data analysis:**

Data from the interviews were analysed thematically.

**Results:**

Four themes were developed: organization of care; diagnosis; support; and views and expectations of the NHS. Patients described cross‐cutting issues such as complex and varied pathways and uncertainty about what would happen next. They felt daunted by the intensity and speed of investigations. They were presented with a recommended course of action rather than options and had little involvement in decision making. They were grateful for care, reluctant to complain and resigned to the status quo.

**Discussion and conclusions:**

In order to meet patient needs, the NHS needs to improve communication and streamline pathways. Future cancer pathways also need to be designed to support shared decision making, be truly person‐centred and informed by patient experience.

## 1 BACKGROUND

Survival rates have improved for many cancers^1^ but remain lower in England than in other developed countries.^2^, ^3^, ^4^, ^5^ Late diagnosis and sub‐optimal access to treatment have been identified as significant drivers of poorer outcomes.^6^, ^7^, ^8^, ^9^, ^10^, ^11^ These findings have encouraged reforms, such as the NHS cancer plan in 2000^12^ that patients in England with symptoms suggestive of suspected cancer should wait no longer than two weeks from urgent GP referral for a specialist appointment and no longer than 62 days from referral to starting treatment, an initiative introduced to reduce waiting times for diagnosis and treatment, minimize psychological distress and save lives.^13^ There is evidence associating this pathway with shorter times to diagnosis and treatment^14^; however, it remains uncertain whether this improves survival.^13^, ^15^, ^16^, ^17^, ^18^, ^19^, ^20^, ^21^

A recent study identified a greater propensity to use referrals for suspected cancer was associated with lower mortality for all cancers combined and for the most common types of cancer.^22^ This supports suggestions that increased primary care use of urgent suspected cancer referrals and associated diagnostic testing may reduce late‐stage diagnoses and mortality of patients with cancer.^23^, ^24^ There is considerable focus on this pathway (and associated cancer targets) from a policy perspective, and almost 40% of cancer patients in England are diagnosed through this route.^25^ However, the study by Round et al (2020)^22^ focused on primary care and GP referrals for suspected cancer but acknowledged the potential for variation once patients are referred, including in the clinical practice of individual specialists, treatments offered and in the wider health‐care system^26^ that are important to consider.^27^ Therefore, there is a need to investigate patient experience of the urgent (two‐week wait) pathway to examine progression through the pathway in greater detail.

Studies suggest there can be similarities in patient experience across cancers.^28^ Whilst we can learn from other types of cancer pathways, the contrasting accounts of pathways described in previous studies also illustrate the diversity in patient experience^29^ and the need to investigate the specific details within a pathway. Existing qualitative research into patient experience of the upper GI cancer referral pathway is minimal.^30^, ^31^, ^32^ Generally, research into patient experience often highlights patient‐related factors. In contrast, factors related to potential improvements that could be made within the health‐care system remain relatively under investigated.^33^ Therefore, there is an imperative to investigate the specific patient experience for upper GI cancer patients in the UK.

Patients with upper gastrointestinal (UGI) cancer have some of the poorest outcomes and longest intervals between referral and commencement of treatment amongst all cancers in England.^34^ In 2018, only 71% of patients with a UGI cancer diagnosis in England had started treatment within the recommended 62 days of referral.^34^ A more efficient pathway could reduce delays, and the considerable variation that currently exists, hence improving equity and, given timeliness is a key feature of high‐quality health care, could improve quality of care and, by implication, increase chances of meeting cancer targets.^35^

One approach to system transformation is to engage patients throughout pathway redesign and implementation to ensure that changes benefit patients in terms of clinical outcomes and experiences of care.^34^ Evidence shows that people have better experiences and improved health and well‐being if their care is personalized and they can actively shape their care and support.^36^, ^37^ Despite evidence supporting the effectiveness of person‐centred care, patient surveys over the past decade have consistently shown that person‐centred care has not been implemented ‘at scale’ and is difficult to do.^38^, ^39^ Recently, the NHS Long Term Plan^37^ has stated that the roll out of the NHS Comprehensive Model for Personalised Care^40^ means patients will get more control over their own health and more personalized care when they need it.

Despite policy rhetoric on the importance of person‐centred care in care pathway development, research on UGI cancer patient experience within the referral pathway remains limited^30^, ^31^, ^32^ and factors related to potential improvements that could be made within the health‐care system remain under‐investigated.^33^ The current study therefore aimed to explore patients’ experiences of the NHS UGI urgent cancer care pathway to identify potential areas of service improvement.

## 2 MATERIALS AND METHODS

### 2.1 Methodological approach

Qualitative semi‐structured telephone interviews were conducted. The study was carried out in accordance with the consolidated criteria for reporting qualitative research (COREQ)^41^ (COREQ checklist included within supporting information S1) and guidance on standards for reporting qualitative research findings.^42^

### 2.2 Participant Recruitment

Patients were eligible if they had initially been referred through the urgent (two‐week wait) GP referral route in the North East and North Cumbria Region of England. The North East comprises 4.6% of the population within the UK,^43^ with a population of 3 187 000 across the North East and North Cumbria Integrated Care System.^44^ It has a mixture of rural and urban areas. The urban areas in particular contain areas of significant deprivation.^45^ Approximately 94% of people resident in the region classified themselves as White British.^46^ Patients were within six months of their first treatment for UGI cancer. Patients referred through any alternative, none of the two‐week pathway was excluded from the study. Clinical Nurse Specialists (CNSs) identified and approached eligible patients. If the patient agreed, the CNS completed a referral form allowing application of purposive sampling (age, sex, area of residence and treatment) of patients. Patients were then given time to consider their involvement while the CNS forwarded the patient's information to the research team. Patients were contacted after a few days to ask whether they still wished to take part in the study and, if so, to arrange an interview at a time convenient to them. Written informed consent was given by each participant before the interview was conducted. Patients were informed that they could withdraw at any time and that this would not affect their care. For confidentiality reasons, the care team (CNSs) were not informed as to whether the patient took part in the study. All participants were given a unique participant number, which allowed results to be anonymous. Patients were informed that anonymous, direct quotes could be used in published materials. The protocol stated that, if a patient became distressed the researcher would stop the interview, allow the patient some time, and then discuss with them whether/how they would wish to proceed; this did not occur. The patients were also advised to contact their cancer nurse specialists if participating in the study had raised by questions about their cancer care.

### 2.3 Ethical approval

The study was classified as service evaluation by NHS HRA and reviewed and approved by the Newcastle University Faculty of Medical Sciences Research Ethics Committee (ref 1272/13714/2017).

### 2.4 Data collection

All interviews (except one, which was face‐to‐face at the interviewee's request) were conducted by one researcher (AH) via telephone, audio‐recorded and transcribed verbatim. Interviews ranged between 20 and 60 minutes. Telephone interviews are an efficient way to collect data. In addition, they can improve participation of more marginalized groups, ease any participant discomfort, and the perception of greater anonymity can lead to greater disclosure.^47^ They were supported by a topic guide (informed by a rapid literature review) (supporting information S2). The areas covered by the topic guide included seeking medical advice, the diagnostic process, information provision and treatment options. The topic guide was used flexibly, to let patients describe their experience in a way that was natural to them, and evolved as recruitment progressed, to enable emerging issues to be explored.

The researcher collecting data was a female health psychologist, PhD, with no role in the care of patients. She has training and previous experience in qualitative methods, particularly interviewing.

### 2.5 Data analysis

Thematic analysis was used to analyse the data. A social constructionism/relativist, semantic and descriptive approach was used. Inductive coding allowed themes to emerge from the data without prior categorization.^48^ Thematic analysis was undertaken in parallel with data collection and used alongside field notes, so that the content of early interviews informed later ones, thus ensuring sufficient depth and that data saturation was reached.^49^ Data saturation was defined using the principle outlined by Francis et al (2009)^50^ ‘*Stopping criterion ‐ After 10 interviews, when three further interviews have been conducted with no new themes emerging, we will define this as the point of data saturation’*. (P1234).

NVivo version 11 software was used to support coding and analysis.^51^ One researcher (AH) coded all interview transcripts, with 20% (n = 4) independently coded by a second researcher (SS). The coding framework (supporting information S3) was then discussed, agreed within the full research team and applied to the remainder of the interview transcripts. Illustrative quotes have been provided to supplement narrative descriptions of the findings.

After development of the themes, the findings were interpreted by the researchers to provide recommendations for future service improvement (Appendix 1).

## 3 RESULTS

Twenty‐seven patients were referred to the research team; four declined and three could not be contacted. Twenty patients (74% of referrals) were interviewed. All interviewees were white British, with 19 men and one woman. The mean age was 66 years (range 48‐77).

Four themes were developed: organization of care; diagnosis; support; and views and expectations of the NHS. The themes included negative and positive aspects of care.

### 3.1 Theme 1: Organization of care

Patients described complex and varied pathways. They were uncertain about what would happen next or how long things would take. Particular issues related to immediate access to testing (endoscopy) versus specialist referral, referral across numerous hospital sites and expectations/communication of waiting times.

Most patients were offered immediate access to testing, but others required a specialist appointment first, which was unexpected.

‘I thought I would then receive an appointment to go straight to the endoscopy department. I didn’t, I received a letter saying that I had an appointment with a consultant gastroenterologist…the consultant said that she is the one that decides whether I need an endoscopy, not the GP’. (Participant 14)

A few patients described short and simple pathways. Others had long and complex pathways, attending multiple appointments in multiple hospitals, often considerable distances apart. On average, patients had eight health‐care appointments (range 4 ‐ 12) from referral to diagnosis. On average, they visited four different hospital sites (range 3 ‐ 6) from referral to initial treatment. It was unclear to patients why there were so many visits.

‘Trying to keep track of where you’re supposed to be and when… I mean, it’s the time span in between your appointments. There was a two‐week time span, for example, where each of those two weeks I was in three different hospitals. One visit involving overnight stay’. (Participant 13)

Patients often felt daunted by the initial intensity and speed of investigations.

‘…of course time is of the essence with cancer. I’m fully aware of that. However, when you’re on the receiving end of all of these appointments, it’s absolutely daunting…They just seem to come at you one after the other, after the other, after the other. And as I say, you can’t argue because time is of the essence. But that still doesn’t stop you feeling overwhelmed by it all’. (Participant 13)

However, although patients described feeling as though they were ‘hardly away from the place [hospital]’, there was a tension between the experience being daunting and the desire to ‘get it sorted’ as soon as possible.

‘….. I thought yes, get on with it. I was very optimistic and said look, let’s just get stuck in and get it sorted as soon as we can, you know? So although it was intense I’d rather have that than it dragged out, here and a bit there, you know, just get on and get the whole thing done and dusted as soon as we can and get moving on’ (Participant 10)

Patients were often unclear about the timescale of the journey ahead and what would happen next.

‘It was all this waiting in between times, wondering what was happening. Just waiting for somebody to say you’ve got two weeks to live [laughs]. It’s all too late’. (Participant 9)

‘I did turn around to him and I did say, “Look, Mr [NAME], it’s April now, this cancer has been growing inside me since January. It was diagnosed in February. We are now into April, and still nobody has done anything to try and knock the cancer back?”’ (Participant 14)

### 3.2 Theme 2: Diagnosis

Patients described how processes and communication around diagnosis caused difficulties. Particular issues related to appropriate delivery of results, prior expectations of the purpose of visits, lack of clarity on timescales, communication and information personalized to patient needs.

Patients spoke about professionals using a range of terms, including cancer, biomass, lesion and tumour, when communicating the initial diagnosis. Several patients said they never heard the word cancer.

‘Yes, after the endoscopy, the sister told me there was a lesion in the oesophagus’. (Participant 9)

Others described how their cancer diagnosis was explained through detailed understandable information.

‘…everything was put in layman’s terms so that I could understand. But the way it was put, and it was put to my wife as well, because she was there, it was exactly what I wanted it to be’. (Participant 4)

However, some patients left hospital with a lack of knowledge of cancer, or their tests results and/or what they meant, and this caused anxiety.

‘I wasn’t aware of what it involved and on reflection I thought that was not pleasant and it wasn’t pleasant on top of what the problem was so it was an added layer of anxiety in reality’. (Participant 2)

Patients contrasted the ‘urgency’ suggested by the requirements placed upon them (and therefore often upon family and friends) to attend numerous appointments, at short notice, in close succession, often across multiple hospital sites, with the seemingly long time taken to receive results, and the unsettling time spent waiting and ‘not knowing’ the results and prognosis.

‘There seems to be an eight to ten day period for any test results to come back. I thought, hang on a second. I don’t understand. If it’s so urgent that I am there, to have it done on the day, there and then, what happens after you’ve had it done? Then the timescale suddenly becomes immediate, or eight to ten days. I didn’t quite understand that’. (Participant 2)

Patients described the importance of clear, consistent, sensitive and jargon‐free communication.

Most patients had surgery and chemotherapy, but a few had radiotherapy and some were on a palliative pathway. Patients described being presented with a recommended course of action, rather than options, with little evidence of patient involvement in shared decision making (SDM).

‘Yes. I think it was really [CNS name] when she rang she said, “Right, we’ve got a plan” and that’s when she laid down, you know, it’s the ECX chemo. Right, it was on the phone she said we have a plan and I think it was a Friday and we went down on the Monday or something, and we discussed it there to, sort of, give us the details’. (Participant 10)

‘So, I mean, the MDT meeting is really, you know, always appeared off limits. I have got another friend who has got cancer, and you know, he has the same issue. You don't know whether someone is saying well, maybe you could do that, and then they decide on balance, no. I mean, you get no choice. You get a, this is what we are going to do. And, unless you have the wherewithal…the knowledge to ask questions, you might not necessarily know what might have been the other options that were discussed’. (Participant 11)

### 3.3 Theme 3: Support

Support from clinical nurse specialists was valued, as was involvement of family and friends. Patients stated how communication style, with careful consideration of the balance between straight talking and sensitivity, and being treated with dignity and respect were important. Patients felt the professionals were working in their best interests to get the best results possible. First impressions of health‐care professionals were important to patients, with the need for professionals to introduce themselves.

‘That was absolutely atrocious, because there was no, he didn’t introduce himself; he didn’t say what I was there for. He had a nurse auxiliary in the room who just kept shaking her head’. (Participant 7)

‘The treatment I have had at the [hospital]. It’s pretty routine for them, I suppose. I felt a bit of a number there, rather than anything else’. (Participant 8)

Many patients viewed the process as daunting but found reassurance from friendly faces.

‘I can only commend the care that I’ve had from start to finish. To say one thing, the most important thing about your care is a smile. One of the things when I was in hospital, was the fact that when you wanted anything, the first thing you got when the nurses or sisters came over, was a smile. You’re off to a good start, anyway’. (Participant 2)

Patients described how family and friends played a crucial role, for example in keeping track of appointments or results and following them up when they were not received.

‘Good job I've had my wife because I would have been lost with the appointments and what have you. She's been a rock for me, organising this and organising‐ of course I don't drive so she does all the driving’. (Participant 16)

‘My wife was kicking the wall, wanting information off people and this that and the other, but I’m sort of pretty easy going, you know’. (Participant 5)

### 3.4 Theme 4: Views and expectations of the NHS

Patients often thought that their personal experience was common practice and they were getting the same as everyone else. This was often a barrier to them suggesting potential improvements for the pathway as they were resigned to the status quo.

‘I mean, obviously you can’t pick and choose, you know, it’s wherever the facilities are really. It’s not a major problem it’s just the way the things are, you know?’ (Participant 20)

‘At the end of the day, they’re trying to save your life and you’re trying to help them save your life, so whatever they want you to do, you do. You go ahead with it…’ (Participant 17)

Patients described feeling grateful for the NHS and felt that staff were time‐pressured but working in their best interests. They spoke about not wanting to be seen as ‘complaining’ and were reluctant to express concerns about health‐care professionals during the interviews.

‘As I say, I know GP’s are very busy people and he probably just gone onto his next patient and was doing the referral and then forget that I needed to make the appointment. So, I as I say, it was much my fault as it was his, I suppose. I’m not going to blame the doctor’. (Participant 9)

‘Well, it’s just waiting times. You understand I’m not complaining here? It’s just an observation. I had to be in for half past seven in the morning and they kept me in the waiting room, and it was 4 o’clock when I went down for my operation… It’s not a complaint, you understand, I’m just letting you know, you know what I mean, what’s happening? I’m not complaining here’ (Participant 19)

The interview findings allowed a range of cross‐cutting issues to surface from our themes. All findings (including these cross‐cutting issues) were used to inform the researchers’ recommendations for future service improvement (Appendix 1).

## 4 DISCUSSION

### 4.1 Statement of principal findings

Timeliness of care is an international challenge. Achieving cancer‐waiting targets within the NHS is one example of this. A further challenge is delivering more patient‐centred care and the inherent conflict between these two NHS policies. Furthermore, an uncompromising focus on pathway expediency may in some cases be detrimental to patient experience of care; for example patients may need time to make important treatment decisions. This study exposed wide variations in the lived experience of the UGI cancer pathway. Whilst several patients reported short and simple pathways, most described complex and varied pathways with numerous appointments, at short notice, in close succession across different organizations and multiple hospital sites, coupled with long waits for test results. Patients were not clear about what would happen next or how long things would take. They often felt underprepared for the intensity and speed of tests, and the long pathway ahead of them. They described being presented with a recommended course of action rather than treatment options, and there was little evidence of involvement of patients in decision making. Patients were grateful for the care received, reluctant to complain and resigned to the status quo.

### 4.2 Strengths and limitations of the study

A patient representative was included in the steering group to ensure all patient‐facing materials (eg information sheet) and the interview topic guide made sense, was sensitive and appropriate to context. Patients were interviewed within six months of their first treatment, thus ensuring that their recall of the events was likely to be reasonably fresh. They lived in different parts of the region, ranged from 48 to 77 years and had had a variety of treatments. They also varied with respect to the number of hospitals they had visited (ranging from 3 to 6) and the number of appointments they had received during their care (ranging from 4 to 13).

The independence of the researcher to the patient's clinical care setting was also a strength as it provided assurance, alongside steps taken to preserve participant anonymity that any remarks made would not impact on the care the person subsequently received, thereby enabling participants to feel they could speak freely without judgement.

One limitation was the lack of female participants interviewed. UGI cancer is more often diagnosed in males (two thirds), but the proportion is still lower than expected and may have impacted on findings. The study lacked ethnic diversity among participants (all participants were white British). In part, this reflects the ethnic diversity within the study area. Previous research has identified ethnic minority cancer patients as reporting lower satisfaction, less positive experiences of care overall and less understanding of health‐care professionals.^52^ This has implications for ethnic inequalities in health care and the study misses the opportunity to uncover these. Further research would be valuable to explore whether differences in experiences of the urgent (two‐week wait) UGI pathway are present in people of different ethnic groups. The interviews were conducted with one cancer specific group, within one timeframe/stage of their care. However, it has been noted that there are many similarities in patient experience across cancers ^28^; therefore, the emergent themes may be applicable to other cancers. The cross‐sectional design (when all participants were recalling what had occurred to them) may be limited by issues such as cognitive dissonance and current health status. However, the diverse experiences reported provides confidence in the data not being entirely based on favourable recall of what happened. Future research using a longitudinal study, tracking people prospectively through pathway (indeed the different pathways), would be valuable. Only including one region in England is another limitation; however, experiences are unlikely to be much different in the North East of England than elsewhere in the country and the participants were from a range of services differing geographically and in terms of organization.

Another potential limitation was the use of CNSs, familiar with the patients, in the recruitment process; this may have biased participation, for example if the CNS only approached patients to participate who they felt held positive views. However, the interviews identified diverse experiences and views and therefore suggest CNS did not solely approach patients with particular views.

### 4.3 Comparison to previous research

Organization of care within the present study suggested efficient communication between primary and secondary care was vital. A potential contributor to patient delays identified in previous research is waiting to have appointments with specialist doctors or for specialist tests.^33^, ^53^, ^54^, ^55^, ^56^, ^57^ In this study, a pathway variation was apparent, with some patients gaining direct access to testing, whilst others were surprised by initial specialist appointments before being sent for tests. This has also been identified in an endoscopy patient experience study^58^ and highlights a potential area for improvement, as previous literature has identified that a straight‐to‐test protocol results in a reduction in times to cancer diagnosis and cancer treatment.^59^, ^60^ In addition, GP direct access testing performed as well as, and on some measures better than, consultant‐triaged testing on measures of disease detection, appropriateness of referrals, interval from referral to testing, and patient and GP satisfaction.^61^

At the referral stage, whilst health‐care professionals may not want to create unnecessary anxiety for patients, previous research suggests fear can actually be increased if the patient feels unclear or unprepared for the cancer referral pathway.^62^, ^63^ Patients in a previous head and neck cancer study reported that health‐care professionals rarely used the word ‘cancer’.^64^ Our study also identified several patients were never diagnosed using the word cancer, often leaving hospital unsure about their diagnosis and what to expect next in their care. This study revealed that even simple communication, such as each clinician introducing themselves or clinicians involving family and friends who were present, could affect the patient's experience when they are amidst a frantic appointment schedule. This links to previous research which suggested training for staff who have to break bad news to patients.^55^ In a complex area such as UGI cancer, where symptoms are often unspecific and pathways variable, clarity, accuracy and honesty of information communication are critical.

Continuity of care was identified in previous literature as important to patients; however, this differed depending on where patients lived and what they were accustomed to.^53^ Continuity of care was seen to be important for knowing who to contact if they were unsure about their care and also in terms of trusting decisions about their treatment.^31^, ^32^, ^53^, ^55^, ^56^, ^57^ The current study confirmed this, with patients describing the importance of being assigned a clinical nurse specialist. However, continuity of care was difficult with other health‐care professionals, as patients were often referred to numerous hospitals along the pathway.

Delays have been attributed to symptom investigation and the primary‐secondary interface.^65^ Previous studies revealed the key factors that patients perceived as impacting on their diagnosis were in the investigation stage.^33^, ^53^, ^54^, ^55^, ^56^, ^57^, ^66^ This study provides further insight into how diagnostic delay is complex and multifaceted, with patient pathways varying in relation to the number of hospitals they visited, appointments they attended and waiting times for test results. The current pathway is embedded in the concept of it being important to diagnose and treat cancer quickly, even though the clinical evidence shows uncertainty whether those who are treated more quickly have better survival.^15^, ^16^, ^17^, ^18^, ^19^, ^20^, ^21^ The urgent referral pathway is giving a message to patients that rapid referral is important. Indeed, within this study, the fact they had been urgently referred established, for patients, the belief that it is important to act quickly to treat cancer. However, patients felt they were provided with a mixed message when the pathway would suddenly slow down and they were left waiting for tests, heightening anxiety rather than reducing it.

Guidance recommends that patients are involved in the decision‐making process,^37^, ^67^, ^68^ with services responding to what matters to the individual, empowering patients to be involved in choices about options for their care.^69^, ^70^ Patients from other cancer pathways have previously described having minimal involvement in decision making with regard to their care ^53^, ^62^, ^71^ and there is a particular challenge with multidisciplinary team decision making.^72^ The present study supports this previous research, with patients stating that they were presented with treatment recommendations rather than options, with little evidence of involvement in decision making. In a recent National Cancer Patient Experience Survey, 21% of all cancer patients said they wanted more involvement in decisions about their care and treatment, with only 35% stating they were given a care plan.^73^ Having an incongruent treatment decision‐making experience has been associated with lower health‐related‐quality‐of‐life (HRQoL) among survivors. These previous findings suggest that involving patients in treatment decisions to the degree to which they want to be involved may help improve cancer survivors' HRQoL.^74^ This illustrates the further need for pathways to consider how patients are supported so they are involved in decision making. It has been acknowledged that the NHS also needs a more fundamental shift in how it works alongside patients and individuals to deliver more person‐centred care.^70^ Creating genuine partnerships requires professionals to work differently, as well as a systematic approach to engaging patients in decisions about their health and well‐being.^37^

The media portray the NHS as in crisis, with hospital specialities usually illustrated as under pressure.^75^ Patients within this current study perceived health‐care professionals to be time‐pressured but working in their best interests. As a result, patients were often reluctant to comment on their care, criticize, complain or provide negative feedback, as they believed staff were doing the best they could in the circumstances. However, patients’ gratitude for the NHS, stoic acceptance of the status quo and reluctance to suggest improvements should not be seen as justification for maintaining the status quo, which will fail to produce the transformations required within health care,^76^ including achieving cancer waiting time targets and ensuring person‐centred care throughout the pathways.

The value of this study is in shedding light on the urgent (two‐week wait) pathway, identifying the specific variation once patients are referred, including in the clinical practice of individual specialists, treatments offered, and in the wider health‐care system,^26^ information that was previously stated as lacking and acknowledged as important to consider.^27^

### 4.4 Recommendations for policy and practice

From the issues raised, a list of robust, evidence‐based findings and recommendations have been identified by the research team to support future service improvement. Many of the recommendations centre on the need for clearer communication with patients. This relates to improvement in the explanation of the pathway ahead, so patients have clear expectations of appointments, results and timescales. This extends to engaging patients, and family and friends when patients agree, in treatment decision making.

### 4.5 Recommendations for research

Within this paper there has not been the opportunity to report every detail of the interview findings. The primary purpose was to explore experiences and therefore issues like decision making are worthy of further detailed exploration and are identified as an area for further research. Further research into the experience of UGI patients throughout their cancer care pathway would be useful within other health‐care systems which have undergone system transformation, such as the Danish model,^77^ to attempt to reduce waiting times and/or improve patient experience and outcomes. There is increasing emphasis on the use of co‐production/co‐design within the NHS and there is strong importance placed on including patient voice within service improvements.^68^ This has been highlighted as an challenging area and demonstrates how further work is needed to shift the balance of power and to give patients agency to realize that raising quality improvement points will lead to action to improve services not only for themselves but for others.^68^, ^70^ Therefore, future research investigating how co‐design, with both health‐care professionals and patients, could be effectively incorporated within the cancer care pathways is vital.

## 5 CONCLUSION

In order to meet patient needs, the NHS needs to improve communication and streamline pathways. Future cancer pathways also need to be designed to support shared decision making, be truly person‐centred and informed by patient experience.

## CONFLICT OF INTEREST

The authors declare no conflict of interest.

## AUTHORS' CONTRIBUTIONS

SS conceived the study with ML, RT and LS. AH conducted the interviews. Data analysis was conducted by AH and SS; other authors contributed to interpretation. AH drafted the manuscript, and all authors read and provided feedback on previous versions. All authors read and approved the final manuscript. SS is guarantor for the research.

## CONSENT FOR PUBLICATION

All participants provided informed consent to publish the findings from the study. All data included in the manuscript are anonymized.

## AVAILABILITY OF DATA AND MATERIALS

The data collected and analysed during the current study are available from the corresponding author on reasonable request.

## ETHICS APPROVAL AND CONSENT TO PARTICIPATE

The study was approved by the Newcastle University Faculty of Medical Sciences Research Ethics Committee (ref 1272/13714/2017), and informed consent was obtained from all participants. The study was performed in accordance with the Declaration of Helsinki.

## Supporting information

Supplementary Material

Supplementary Material

Supplementary Material

## Appendix 1 Service improvement recommendations (for each interview theme)

### Theme 1 ‐ NHS systems and pathways/organization of care

Recommendation – Pathways should be reviewed to reduce unwarranted variation and to simplify the patient's journey.

Clear communication is a necessary component but is insufficient in the absence of a review of the pathway.

Recommendation – Clearer communication with patients to

- Ensure they understand the pathway they are entering
- Ensure they understand throughout what the next steps are (results, feedback).

### Theme 2 ‐ diagnosis

Recommendation – improve communication with patients to:

- support clear expectations for each visit (patient information),
- provide person‐centred consultations and explain timescales for next steps (consultation skills),
- provide results to patients through the appropriate channel (eg face to face with a clinician) and
- review the process of engaging patients in shared decision making.

### Theme 3 – support

Recommendation – improve communication with patients by:

- clinicians introducing themselves (#mynameis),
- sensitive but honest presentation of results (consultation skills) and
- appreciating the importance of family and friends.

### Theme 4 ‐ Views and expectations of the NHS

Recommendation – patients’ stoic acceptance of the status quo should not be seen as justification for maintaining the status quo.

Recommendation – there is a need to actively capture reliable and valid patient feedback on both positive and negative experiences of care to inform quality improvement. This could be enhanced by:

- A review of the current methods and approaches to obtaining patient feedback, with consideration of independent (eg conducted by people outside of those delivering the service) and mixed methods (eg surveys, interviews and workshops) approaches.
- Clinicians and managers enabling and welcoming staff feedback and comment.

## References

[1] Quaresma M , Coleman MP , Rachet B . 40‐year trends in an index of survival for all cancers combined and survival adjusted for age and sex for each cancer in England and Wales, 1971–2011: a population‐based study. Lancet. 2015;385(9974):1206‐1218. 25479696 10.1016/S0140-6736(14)61396-9

[2] Coleman MP , Forman D , Bryant H , et al. Cancer survival in Australia, Canada, Denmark, Norway, Sweden, and the UK, 1995–2007 (the International Cancer Benchmarking Partnership): an analysis of population‐based cancer registry data. Lancet. 2011;377(9760):127‐138. 21183212 10.1016/S0140-6736(10)62231-3 PMC3018568

[3] De Angelis R , Sant M , Coleman MP , et al. Cancer survival in Europe 1999–2007 by country and age: results of EUROCARE–5‐a population‐based study. Lancet Oncol. 2014;15(1):23‐34. 24314615 10.1016/S1470-2045(13)70546-1

[4] Walters S , Benitez‐Majano S , Muller P , et al. Is England closing the international gap in cancer survival? Br J Cancer. 2015;113(5):848‐860. 26241817 10.1038/bjc.2015.265 PMC4559829

[5] Arnold M , Rutherford MJ , Bardot A , et al. Progress in cancer survival, mortality, and incidence in seven high‐income countries 1995–2014 (ICBP SURVMARK‐2): a population‐based study. Lancet Oncol. 2019;20(11):1493‐1505. 31521509 10.1016/S1470-2045(19)30456-5 PMC6838671

[6] Dejardin O , Rachet B , Morris E , et al. Management of colorectal cancer explains differences in 1‐year relative survival between France and England for patients diagnosed 1997–2004. Br J Cancer. 2013;108:775. 23392081 10.1038/bjc.2013.33 PMC3590669

[7] Maringe C , Walters S , Butler J , et al. Stage at diagnosis and ovarian cancer survival: evidence from the International Cancer Benchmarking Partnership. Gynecol Oncol. 2012;127(1):75‐82. 22750127 10.1016/j.ygyno.2012.06.033

[8] Maringe C , Walters S , Rachet B , et al. Stage at diagnosis and colorectal cancer survival in six high‐income countries: a population‐based study of patients diagnosed during 2000–2007. Acta Oncologica. 2013;52(5):919‐932. 23581611 10.3109/0284186X.2013.764008

[9] Walters S , Maringe C , Butler J , Brierley JD , Rachet B , Coleman MP . Comparability of stage data in cancer registries in six countries: lessons from the International Cancer Benchmarking Partnership. Int J Cancer. 2013;132(3):676‐685. 22623157 10.1002/ijc.27651

[10] Walters S , Maringe C , Butler J , et al. Breast cancer survival and stage at diagnosis in Australia, Canada, Denmark, Norway, Sweden and the UK, 2000–2007: a population‐based study. Br J Cancer. 2013;108:1195. 23449362 10.1038/bjc.2013.6 PMC3619080

[11] Walters S , Maringe C , Coleman MP , et al. Lung cancer survival and stage at diagnosis in Australia, Canada, Denmark, Norway, Sweden and the UK: a population‐based study, 2004–2007. Thorax. 2013;68(6):551‐564. 23399908 10.1136/thoraxjnl-2012-202297

[12] Department of Health . The NHS Cancer Plan. A plan for investment, a plan for reform. In: 2000.

[13] Møller H , Gildea C , Meechan D , Rubin G , Round T , Vedsted P . Use of the English urgent referral pathway for suspected cancer and mortality in patients with cancer: cohort study. BMJ. 2015;351:h5102. 26462713 10.1136/bmj.h5102 PMC4604216

[14] Neal RD , Din NU , Hamilton W , et al. Comparison of cancer diagnostic intervals before and after implementation of NICE guidelines: analysis of data from the UK General Practice Research Database. Br J Cancer. 2014;110(3):584‐592. 24366304 10.1038/bjc.2013.791 PMC3915139

[15] Aslam MI , Chaudhri S , Singh B , Jameson JS . The "two‐week wait" referral pathway is not associated with improved survival for patients with colorectal cancer. Int J Surg. 2017;43:181‐185. 28600231 10.1016/j.ijsu.2017.05.046

[16] Currie AC , Evans J , Smith NJ , Brown G , Abulafi AM , Swift RI . The impact of the two‐week wait referral pathway on rectal cancer survival. Colorectal Dis. 2012;14(7):848‐853. 21920010 10.1111/j.1463-1318.2011.02829.x

[17] Fallon M , Adil MT , Ahmed K , Whitelaw D , Rashid F , Jambulingam P . Impact of ‘two‐week wait’ referral pathway on the diagnosis, treatment and survival in upper and lower gastrointestinal cancers. Postgrad Med J. 2019;95(1127):470‐475. 31147396 10.1136/postgradmedj-2019-136507

[18] Palser TR , Cromwell DA , Hardwick RH , Riley SA , Greenaway K , van der Meulen JHP . Impact of route to diagnosis on treatment intent and 1‐year survival in patients diagnosed with oesophagogastric cancer in England: a prospective cohort study. BMJ Open. 2013;3(2):e002129. 10.1136/bmjopen-2012-002129 PMC3585975 23408076

[19] Sharpe D , Williams RN , Ubhi SS , Sutton CD , Bowrey DJ . The "two‐week wait" referral pathway allows prompt treatment but does not improve outcome for patients with oesophago‐gastric cancer. Eur J Surg Oncol. 2010;36(10):977‐981. 20702059 10.1016/j.ejso.2010.07.002

[20] Wong BYW , Fischer S , Cruickshank HE . Clinical outcome of head and neck cancer patients: a comparison between ENT patients referred via the 2 weeks wait pathway and alternative routes in the UK health system. Eur Arch Otorhinolaryngol. 2017;274(1):415‐420. 27416743 10.1007/s00405-016-4200-5

[21] Zafar A , Mak T , Whinnie S , Chapman MA . The 2‐week wait referral system does not improve 5‐year colorectal cancer survival. Colorectal Dis. 2012;14(4):e177‐e180. 21920007 10.1111/j.1463-1318.2011.02826.x

[22] Round T , Gildea C , Ashworth M , Møller H . Association between use of urgent suspected cancer referral and mortality and stage at diagnosis: a 5‐year national cohort study. Br J Gen Pract. 2020;70(695): e389‐e398. 32312762 10.3399/bjgp20X709433 PMC7176359

[23] Hamilton W , Walter FM , Rubin G , Neal RD . Improving early diagnosis of symptomatic cancer. Nat Rev Clin Oncol. 2016;13(12):740‐749. 27458007 10.1038/nrclinonc.2016.109

[24] Hamilton W . Diagnosing symptomatic cancer in the NHS. BMJ. 2015; 351: h311. 10.1136/bmj.h5311 26466605

[25] National Cancer Registration and Analysis Service . Routes to Diagnosis. http://www.ncin.org.uk/publications/routes_to_diagnosis Accessed 1st July 2020. Published 2016, Accessed

[26] Møller H , Coupland VH , Tataru D , et al. Geographical variations in the use of cancer treatments are associated with survival of lung cancer patients. Thorax. 2018;73(6):530‐537. 29511056 10.1136/thoraxjnl-2017-210710 PMC5969334

[27] Burton C , O’Neill L , Oliver P , Murchie P . Contribution of primary care organisation and specialist care provider to variation in GP referrals for suspected cancer: ecological analysis of national data. BMJ Qual Saf. 2019;29(4):296‐303. 10.1136/bmjqs-2019-009469 31586938

[28] Weller D , Vedsted P , Rubin G , et al. The Aarhus statement: improving design and reporting of studies on early cancer diagnosis. Br J Cancer. 2012;106(7):1262‐1267. 22415239 10.1038/bjc.2012.68 PMC3314787

[29] Black G , Sheringham J , Spencer‐Hughes V , et al. Patients’ Experiences of Cancer Diagnosis as a Result of an Emergency Presentation: A Qualitative Study. PLoS One. 2015;10(8):e0135027. 26252203 10.1371/journal.pone.0135027 PMC4529308

[30] Andreassen S , Randers I , Ternulf Nyhlin K , Mattiasson AC . A meta‐analysis of qualitative studies on living with oesophageal and clinically similar forms of cancer, seen from the perspective of patients and family members. Int J Qual Stud Health Well‐being. 2007;2(2):114‐127.

[31] Malmström M , Klefsgård R , Johansson J , Ivarsson B . Patients' experiences of supportive care from a long‐term perspective after oesophageal cancer surgery ‐ A focus group study. Eur J Oncol Nurs. 2013;17(6):856‐862. 23732012 10.1016/j.ejon.2013.05.003

[32] Schildmann J , Ritter P , Salloch S , Beiderwellen P , Vollmann J . Treatment decision making in pancreatic cancer. A qualitative interview study on the views and preferences of patients. Onkologie. 2012;35:225.

[33] Molassiotis A , Wilson B , Brunton L , Chandler C . Mapping patients' experiences from initial change in health to cancer diagnosis: A qualitative exploration of patient and system factors mediating this process. Eur J Cancer Care. 2010;19(1):98‐109. 10.1111/j.1365-2354.2008.01020.x 19552730

[34] NHS England .Implementing a timed oesophago‐gastric cancer diagnostic pathway. 2019.

[35] Agency for Healthcare Reasearch and Quality . Six Domains of Health Care Quality. https://www.ahrq.gov/talkingquality/measures/six‐domains.html. Published 2018. Accessed 7th January, 2019

[36] Lopatina E , Miller JL , Teare SR , et al. The voice of patients in system redesign: A case study of redesigning a centralized system for intake of referrals from primary care to rheumatologists for patients with suspected rheumatoid arthritis. Health Expect. 2019;22(3):348‐363. 30520175 10.1111/hex.12855 PMC6543166

[37] NHS . The NHS Long Term Plan. 2018.

[38] Mathers N , Paynton D . Rhetoric and reality in person‐centred care: introducing the House of Care framework. Br J Gen Pract. 2016;66(642):12‐13. 26719459 10.3399/bjgp16X683077 PMC4684003

[39] Joseph‐Williams N , Lloyd A , Edwards A , et al. Implementing shared decision making in the NHS: lessons from the MAGIC programme. BMJ. 2017;357:j1744. 28420639 10.1136/bmj.j1744 PMC6284240

[40] NHS England . Comprehensive Model for Personalised Care. 2018.

[41] Tong A , Sainsbury P , Craig J . Consolidated criteria for reporting qualitative research (COREQ): a 32‐item checklist for interviews and focus groups. Int J Qual Health Care. 2007;19(6):349‐357. 17872937 10.1093/intqhc/mzm042

[42] O'Brien BC , Harris IB , Beckman TJ , Reed DA , Cook DA . Standards for reporting qualitative research: a synthesis of recommendations. Acad Med. 2014;89(9):1245‐1251. 24979285 10.1097/ACM.0000000000000388

[43] Statistics OfN . Regional ethnic diversity. https://www.ethnicity‐facts‐figures.service.gov.uk/uk‐population‐by‐ethnicity/national‐and‐regional‐populations/regional‐ethnic‐diversity/latest#ethnic‐groups‐by‐area. Published 2019. Accessed

[44] NHS North of England Commissioning Support Unit . Facts and Statistics. https://nhsjoinourjourney.org.uk/what‐we‐are‐doing/facts‐and‐statistics/. Published 2020. Accessed 1st July, 2020

[45] Ministry of Housing Communities and Local Government . The English Indices of Deprivation ‐ Statistical Release. https://assets.publishing.service.gov.uk/government/uploads/system/uploads/attachment_data/file/835115/IoD2019_Statistical_Release.pdf. Published 2019. Accessed

[46] Office for National Statistics . 2011 Census data. https://www.ons.gov.uk/census/2011census/2011censusdata. Published 2011. Accessed

[47] Drabble L , Trocki KF , Salcedo B , Walker PC , Korcha RA . Conducting qualitative interviews by telephone: Lessons learned from a study of alcohol use among sexual minority and heterosexual women. Qual Soc Work. 2016;15(1):118‐133. 26811696 10.1177/1473325015585613 PMC4722874

[48] Braun V , Clarke V . Using thematic analysis in psychology. Qual Res Psychol. 2006;3(2):77‐101.

[49] Ritchie J , Je L . Qualitative research practice: a guide for social science students and researchers. London: Sage Publications; 2003.

[50] Francis JJ , Johnston M , Robertson C , et al. What is an adequate sample size? Operationalising data saturation for theory‐based interview studies. Psychol Health. 2010;25(10):1229‐1245. 20204937 10.1080/08870440903194015

[51] NVivo qualitative data analysis software [computer program]. Version 112015.

[52] Pinder RJ , Ferguson J , Møller H . Minority ethnicity patient satisfaction and experience: results of the National Cancer Patient Experience Survey in England. BMJ Open. 2016;6(6):e011938. 10.1136/bmjopen-2016-011938 PMC4932347 27354083

[53] Bain NSC , Campbell NC , Ritchie LD , Cassidy J . Striking the right balance in colorectal cancer care ‐ A qualitative study of rural and urban patients. Fam Pract. 2002;19(4):369‐374. 12110557 10.1093/fampra/19.4.369

[54] Brousselle A , Breton M , Benhadj L , et al. Explaining time elapsed prior to cancer diagnosis: patients’ perspectives. BMC Health Serv Res. 2017;17(1):448. 28659143 10.1186/s12913-017-2390-1 PMC5490154

[55] Davies E , Van Der Molen B , Cranston A . Using clinical audit, qualitative data from patients and feedback from general practitioners to decrease delay in the referral of suspected colorectal cancer. J Eval Clin Pract. 2007;13(2):310‐317. 17378881 10.1111/j.1365-2753.2006.00820.x

[56] Pascoe SW , Veitch C , Crossland LJ , et al. Patients' experiences of referral for colorectal cancer. BMC Fam Pract. 2013;14:124. 23972115 10.1186/1471-2296-14-124 PMC3765755

[57] Tsianakas V , Maben J , Wiseman T , et al. Using patients' experiences to identify priorities for quality improvement in breast cancer care: patient narratives, surveys or both? BMC Health Serv Res. 2012;12:271. 22913525 10.1186/1472-6963-12-271 PMC3466127

[58] Neilson LJ , Patterson J , von Wagner C , et al. Patient experience of gastrointestinal endoscopy: informing the development of the Newcastle ENDOPREM™. Front Gastroenterol. 2020;11(3):209‐217. 10.1136/flgastro-2019-101321 PMC7223270 32419912

[59] Christopher J , Flint TR , Ahmed H , et al. Straight‐to‐test for the two‐week‐wait colorectal cancer pathway under the updated NICE guidelines reduces time to cancer diagnosis and treatment. Ann R Coll Surg Engl. 2019;101(5):333‐339. 10.1308/rcsann.2019.0022 PMC6513371 31042431

[60] Jones CP , Fallaize RC , Longman RJ . Updated ‘two‐week wait’ referral guidelines for suspected colorectal cancer have increased referral volumes without improving cancer detection rates. BJMP. 2019;12(2):a012.

[61] Smith CF , Tompson AC , Jones N , et al. Direct access cancer testing in primary care: a systematic review of use and clinical outcomes. Br J Gen Pract. 2018;68(674):e594‐e603. 30104328 10.3399/bjgp18X698561 PMC6104856

[62] Banks J , Walter FM , Hall N , Mills K , Hamilton W , Turner KM . Decision making and referral from primary care for possible lung and colorectal cancer: A qualitative study of patients' experiences. Br J Gen Pract. 2014;64(629):e775‐e782. 25452542 10.3399/bjgp14X682849 PMC4240150

[63] Ndukwe N , Jenkins K , Branagan G , King L . The experiences of patients referred for colorectal symptoms on a rapid referral 'two week rule' ‐ An examination of quantitative and qualitative data from two hospitals. Psycho‐Oncology. 2010;19:S171‐S172.

[64] Deane J , Patterson J , Sharp L .“I thought there would have been pain” A qualitative investigation of patients’ experiences of the route to, and diagnosis of, head and neck cancer. Paper presented at: NCRI Cancer Conference 2019; Glasgow.

[65] Williams P , Murchie P , Bond C . Patient and primary care delays in the diagnostic pathway of gynaecological cancers: a systematic review of influencing factors. Br J Gen Pract. 2019;69(679):e106‐e111. 30642909 10.3399/bjgp19X700781 PMC6355279

[66] Howell DA , Hart RI , Smith AG , Macleod U , Patmore R , Roman E . Disease‐related factors affecting timely lymphoma diagnosis: a qualitative study exploring patient experiences. Br J Gen Pract. 2019;69(679):e134‐e145. 30692091 10.3399/bjgp19X701009 PMC6355261

[67] National Institute for Health and Clinical Excellence . Suspected cancer: recognition and referral. NICE guideline [NG12]. In: 2017. 32040285

[68] Independent Cancer Taskforce . Achieving world‐class cancer outcomes: A strategy for England 2015–2020. In: Health Do, ed 2015.

[69] England NHS . Shared commitment to quality from the National Quality Board. 2016.

[70] Sanderson J , Kay N , Watts R . Universal Personalised Care: Implementing the Comprehensive Model. London: NHS England; 2019.

[71] Edmondson AJ , Birtwistle JC , Catto JWF , Twiddy M . The patients’ experience of a bladder cancer diagnosis: a systematic review of the qualitative evidence. J Cancer Surviv. 2017;1‐9. 10.1007/s11764-017-0603-6 PMC5500680 28213769

[72] Hamilton DW , Heaven B , Thomson RG , Wilson JA , Exley C . Multidisciplinary team decision‐making in cancer and the absent patient: a qualitative study. BMJ Open. 2016;6(7):e012559. 10.1136/bmjopen-2016-012559 PMC4964245 27443554

[73] Abt Sacks A , Perestelo‐Perez L , Rodriguez‐Martin B , et al. Breast cancer patients’ narrative experiences about communication during the oncology care process: a qualitative study. Eur J Cancer Care. 2016;25(5):719‐733. 10.1111/ecc.12384 26412025

[74] Drummond FJ , Gavin AT , Sharp L . Incongruence in treatment decision making is associated with lower health‐related quality of life among prostate cancer survivors: results from the PiCTure study. Support Care Cancer. 2018;26(5):1645‐1654. 29222597 10.1007/s00520-017-3994-z

[75] Barry E , Greenhalgh T . General practice in UK newspapers: an empirical analysis of over 400 articles. Br J Gen Pract. 2019;69(679):e146‐e153. 30642907 10.3399/bjgp19X700757 PMC6355271

[76] Goodridge D , Isinger T , Rotter T . Patient family advisors’ perspectives on engagement in health‐care quality improvement initiatives: Power and partnership. Health Expect. 2018;21(1):379‐386. 28960630 10.1111/hex.12633 PMC5750697

[77] Ingeman ML , Christensen MB , Bro F , Knudsen ST , Vedsted P . The Danish cancer pathway for patients with serious non‐specific symptoms and signs of cancer–a cross‐sectional study of patient characteristics and cancer probability. BMC Cancer. 2015;15(1):421. 25990247 10.1186/s12885-015-1424-5 PMC4445271

