# Supplementary material for: Patient experiences of the urgent cancer referral pathway—Can the NHS do better? Semi‐structured interviews with patients with upper gastrointestinal cancer
Source: Health Expect. 2020 Sep 28;23(6):1512–22. doi: 10.1111/hex.13136 (PMC7752202; doi:10.1111/hex.13136)
Supplement: Supplementary file 2 — Supplementary Material [file HEX-23-1512-s002.docx]

Supplementary information 2 – Interview topic guide

1. Discuss format of interview – signpost through patient journey from first seeing your GP, being informed of the cancer, being provided with information and then moving onto details about how your treatment was discussed and decided.
2. Seeking medical advice

Can you tell me what first led you to seek medical advice?

1. Diagnostic process

What happened after you had seen your doctor?

*Checklist to cover:*

1. GP
2. Consultant/Hospital (same hospital, moving between hospitals)
3. Tests
4. Time at various stages
5. Information provision

I appreciate it may be difficult but could you tell me about the type of cancer you have?

What happened after you were told you had cancer?

*Checklist to cover:*

1. Diagnosis
2. Explanation of diagnosis
3. Supporting materials
4. Explanation of materials
5. Treatment

How were treatment options discussed with you once you were informed you had cancer?

*Checklist to cover:*

1. Treatment options
2. Treatment decision
3. Involvement in decision
4. Relationship with health personnel

Tell me about the staff that you have been in contact with through the stages that we have already discussed.

What level of confidence and trust did you have in the staff you came in contact with?

*Checklist to cover:*

1. Health care professionals involved
2. Relationship with HCPs (support)
3. Discussions (privacy)
4. Additional provision of support or education
5. Evaluation of care received

How was your experience of the care you have received?

Did you feel at any stage that you had an unacceptable "wait", from the first time you presented to the end of your treatment? Why did you feel this was unacceptable?

*Checklist to cover:*

1. Actual experience
2. Expectations
3. Co-design

What do you feel about the possibility for patients and staff to work together to improve services in the NHS?

Do you have any other points you would like to tell me that we have not covered already?

(Positive, negative, improvements)

*Checklist to cover:*

1. Co-design
2. Any other issues not discussed
