# Supplementary material for: Patient experiences of the urgent cancer referral pathway—Can the NHS do better? Semi‐structured interviews with patients with upper gastrointestinal cancer
Source: Health Expect. 2020 Sep 28;23(6):1512–22. doi: 10.1111/hex.13136 (PMC7752202; doi:10.1111/hex.13136)
Supplement: Supplementary file 3 — Supplementary Material [file HEX-23-1512-s003.docx]

| Themes | Codes |
| --- | --- |
| Organisation of care | - Symptoms - Referral and diagnostic procedures - Transfer of patient (and notes) to specialist hospitals - Variety of hospital (location, necessary for tests) - Length of time during tests - Volume, speed, intensity, or progression for tests - Use of private providers - The unknown (waiting) |
| Diagnosis | - Explanation of tests OR diagnosis (outlook, understanding) - Provision of information (preparation, language, expectations) - Patient understanding - Reaction to news - Treatment plan/options - Patient involvement |
| Support | - Health care professional: - Level of care - Familiarity - Reassurance - Trust - Confidence - Patient AND family/friends reaction: - Support - Emotion (upset) - Decision making - Understanding the patient |
| Views and expectations of the NHS | - Standard of care - Gratitude for care received - Resignation to status quo - Sharing of experiences - Understanding - Rationale thinking - Perspectives/priorities - Desire to help other patients |

Supplementary information 3 - Coding framework
